# Design of Broadband High-Frequency Multi-Throw RF-MEMS Switches

**DOI:** 10.3390/mi15070813

**Published:** 2024-06-23

**Authors:** Jian Yu, Maoyun Zhang, Jing Li, Yuheng Si, Zijun Zhu, Qiannan Wu, Mengwei Li

**Affiliations:** 1 School of Instrument and Electronics, North University of China, Taiyuan 030051, China; jianyu_nuc@163.com (J.Y.); zmy_study_hard@163.com (M.Z.); lijing@tit.edu.cn (J.L.); 18834372826@163.com (Y.S.); 2 School of Instrument and Intelligent Future Technology, North University of China, Taiyuan 030051, China; 19581548505@163.com; 3 Academy for Advanced Interdisciplinary Research, North University of China, Taiyuan 030051, China; 4 Center for Microsystem Intergration, North University of China, Taiyuan 030051, China; 5 School of Semiconductors and Physics, North University of China, Taiyuan 030051, China; 6 Key Laboratory of Dynamic Measurement Technology, North University of China, Taiyuan 030051, China

**Keywords:** 3P3T, broadband, fast response, tunable, isolation

## Abstract

This paper introduces a broadband triple-pole triple-throw (3P3T) RF MEMS switch with a frequency range from DC to 380 GHz. The switch achieves precise signal control and efficient modulation through its six-port design. It achieves an insertion loss of −0.66 dB across its frequency range, with isolation and return loss metrics of −32 dB and −15 dB, respectively. With its low actuation voltage of 6.8 V and rapid response time of 2.28 μs, the switch exemplifies power-efficient and prompt switching performance. The compact design is ideal for integration into space-conscious systems. This switch is pivotal for 6G research and has potential applications in satellite communications, military radar systems, and next-generation radio applications that require multi-antenna access.

## 1. Introduction

In recent years, as 6G technology has emerged, there has been a growing demand for enhanced communication system performance, particularly regarding frequency coverage. Communication via 6G is expected to encompass a broad frequency range from millimeter wave (mmWave) to terahertz (THz), underscoring the criticality of RF and mmWave technologies in meeting future communication requirements [1,2,3,4]. The 6G era is projected to deliver communication speeds surpassing 5G, reduced latency, and more intricate device interconnectivity [5]. In response to these advancements, microdevices need further miniaturization, reduced power consumption, and support for heterogeneous integration. Consequently, extensive research has been undertaken on large-scale multiple-input multiple-output (MIMO) units [6,7]. In this context, RF Microelectromechanical Systems (RF MEMS) multi-throw switches have proven indispensable due to their capability for low loss, rapid switching, and extensive frequency range coverage [8,9]. Especially as modern communication devices continue to reduce in size at the RF front end, these multi-throw switches have become essential components for delivering diverse communication services [10,11,12]. RF MEMS switches, fabricated using MEMS processing techniques, are designed to replace conventional devices in the traditional RF and microwave domains, leveraging the many benefits of MEMS to enhance various performance parameters of conventional RF and microwave devices, including high linearity, low power consumption, reduced insertion loss, and expanded bandwidth [13,14,15,16].

While past research has achieved significant advancements in this area, each device has inherent limitations [17,18]. Capacitive and ohmic switches represent the two most common categories [19]. Given their specific frequency band constraints, capacitive switches are unsuitable for multi-band coverage, such as 2G to 6G. In contrast, ohmic switches can cover the entire frequency range starting from DC, making them the primary focus of this study.

In recent years, numerous research groups have dedicated their efforts to enhancing the RF characteristics and mechanical properties of RF MEMS. In 2018, Gopalan and Kommuri introduced a small, low-actuation voltage RF MEMS switch with a triangular design suitable for phased-array applications [20]. This switch achieved an isolation of 26 dB below 20 GHz and had an actuation voltage of 5.6 V. Recognizing the increasing demands for higher frequencies in communication systems, in 2022, Feng et al. ventured into the realm of THz MEMS switches. They presented a design spanning a remarkable frequency range from 140 to 750 GHz [21]. Although this design has an insertion loss nearing 3 dB and an isolation of 12 dB, its ability to cover such high frequencies is noteworthy. However, the actuation voltage of 55 V might present challenges for specific applications, especially those aiming for miniaturization in compact communication systems. That same year, Kim et al. examined a monolayer molybdenum disulfide switch tailored for 6G communication systems, operating from DC to 480 GHz and achieving isolation of 13 dB [22]; however, high-frequency signal isolation still requires enhancement. Gandhi et al. designed an SP4T switch with an impressive isolation reaching 62 dB but only operated at 14 GHz [23]. In 2023, Bansal proposed a broadband terahertz MEMS ohmic switch for 6G communication operating at 150 GHz, achieving 20 dB of isolation and an actuation voltage of 20 V, which remains relatively high for integrated devices [24]. In the same year, Rajasekhar et al. showcased an SPMT switch design with a multi-contact cantilever structure, operating at an actuation voltage of 3.5 V but limited to frequencies below 10 GHz [25]. From these studies, it becomes evident that optimization in terms of actuation voltage, insertion loss, and isolation remains a challenge for high-frequency switches. Most studies still focus on single-throw switches at high frequencies, while multi-throw switch research in these bands is limited. As systems become increasingly compact, the demand for multi-throw switches grows, posing a pressing issue to address.

To address this research gap, we present an innovative triple-pole triple-throw (3P3T) RF MEMS switch designed for multi-band coverage in more complex and compact systems. Simulated using the Finite Element Method (FEM), our switch demonstrates superior performance characteristics, including wide bandwidth, low actuation voltage, excellent S-parameters, and rapid response times, making it an ideal candidate for 6G applications, particularly in satellite communications, military radar, and the Internet of Things.

## 2. Structure and Theoretical Analysis

**Figure 1:**
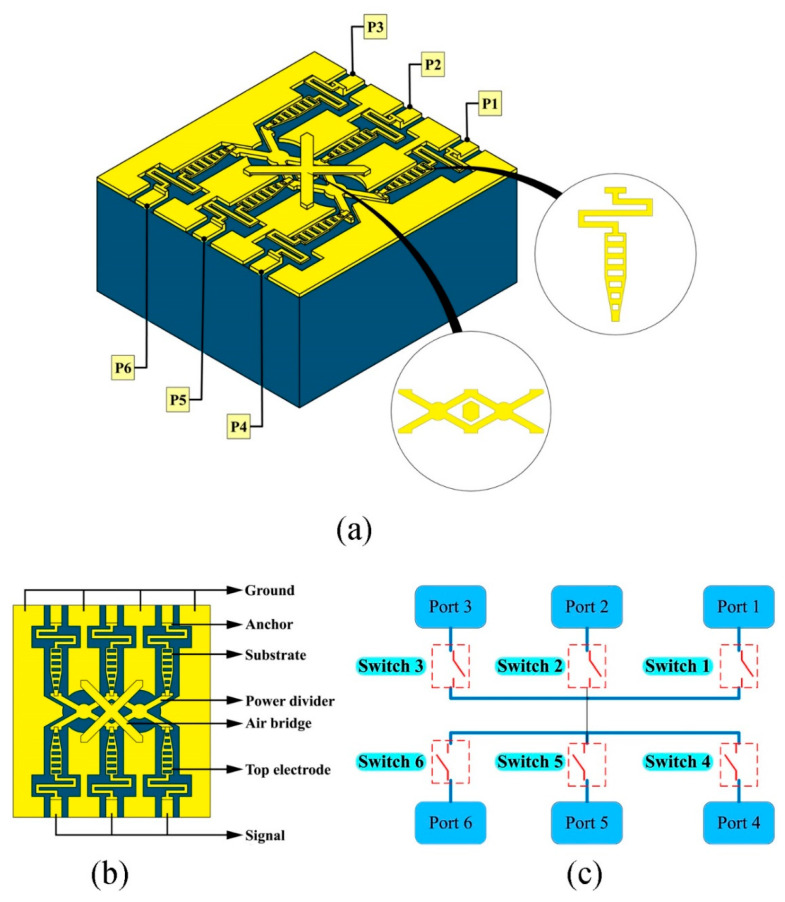
Proposed switch. (**a**) 3D schematic; (**b**) Top view; (**c**) Operating modes.

A three-dimensional schematic of the switch is displayed in Figure 1a, featuring a cantilever beam design with six ports. Connections or isolations between different ports can be achieved by manipulating the driving electrodes. The local view provides a detailed top-view depiction of a single switch and its power divider. The switch comprises several components: signal lines, ground lines, CPW (coplanar waveguide) transmission lines, upper electrodes, anchor points, and a power divider. The power divider ensures an equitable energy distribution from the input port to each output port within this device. An upper electrode design close in width to the transmission line optimizes insertion loss performance. Additionally, the apertures in the upper electrode effectively accelerate the release speed of the beam, which is crucial for the yield of the beam structure and potential large-scale production. Regarding substrate material selection, we opted for a BF33 glass substrate with a dielectric constant of 4.6, given its attributes of low relative permittivity, minimal loss tangent, thermal stability, impact resistance, and high-temperature endurance. The intricate multi-channel signal regulation capability of the switch is demonstrated in Figure 1c. Notably, due to the switch’s holistic symmetric design, any port can be the input, while the remaining ports can function as outputs. Through our optimization studies, each port exhibits exceptional RF performance. Amid the growing scarcity of spectral resources, such designs play a pivotal role in advancing the development of highly integrated and tunable devices. The primary design parameters of the switch are listed in Table 1.

**Figure 2:**
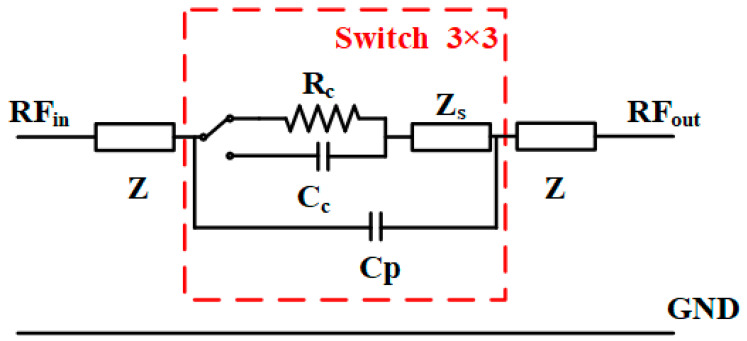
Equivalent circuit of the proposed switch.

The following section provides a detailed analysis of the RF and physical characteristics of the switch. For the ohmic RF MEMS switch, when a sufficient bias voltage is applied between the upper and lower electrodes, the electrostatic force draws the upper electrode downwards, closing the signal. Upon removing the drive voltage between these electrodes, the upper electrode reverts to its original position due to the restoring force, thereby disconnecting the signal. When analyzing the RF MEMS switch’s RF performance, researchers often employ equivalent parameters. In high-frequency circuits, the lumped parameter equivalent circuit can describe the microwave performance of the RF MEMS switch. By establishing its resistance, inductance, and capacitance (*RLC*) model, one can gain a deeper understanding of the effect of each parameter on microwave performance. The switch’s equivalent model is illustrated in Figure 2, where Z represents the characteristic impedance of the CPW signal input/output port. The coupling capacitance of the CPW signal input/output end is denoted as $C_{p}$, and the equivalent resistance of the plane-type upper electrode is represented as $Z_{s}$. The contact resistance of the upper electrode in the closed state is $R_{c}$. When the switch is off, the coupling capacitance generated between the upper electrode and the signal line is $C_{c}$.

Microwave theory is utilized to compute the equivalent circuit results of the RF MEMS switch. The isolation expression obtained in the off state is as follows:

(1) $$S_{21}=20lg\left|\frac{2jwC_{c}Z}{1+2jwC_{c}Z}\right|$$

From this, it can be deduced that the coupling capacitance value between the upper electrode and the signal line significantly influences the isolation of the RF MEMS switch. This study adopts a gradient upper electrode approach to minimize the coupling capacitance value, consequently enhancing the isolation of the RF MEMS switch.

The cantilever beam used in this study has the following fundamental characteristics: the beam’s bending occurs only in the longitudinal direction without involving lateral deformation, and the beam’s deflection is relatively small compared to its length. Based on these characteristics, two fundamental assumptions are proposed: first, under the action of bending moments alone, the beam’s cross-section remains planar during the deflection process, known as the pure bending assumption; second, the beam’s deflection is much smaller than its length, referred to as the small deformation assumption, which allows us to neglect shear stress relative to normal stress. Based on these assumptions, the mechanical deformation of the switch’s top plate can be described using a linear elastic coefficient k.

A pivotal metric for the RF MEMS switch is the drive voltage. An excessively high drive voltage complicates its integration with other devices. Their fundamental vertical movement model consists of two parallel substrates regardless of series contact or parallel capacitive types. The electrostatic force exerted on the upper electrode is as follows:

(2) $$F_{e}=\frac{1}{2}V^{2}\frac{dc\left(g_{0}\right)}{dg_{0}}=-\frac{1}{2}\frac{\varepsilon_{0}AV^{2}}{g_{0}^{2}}$$

Here, $\varepsilon_{0}$ represents the dielectric constant of vacuum or air, A signifies the face-to-face area between the upper electrode and the driving electrode, V denotes the switch’s drive voltage, and $g_{0}$ is the gap between the upper electrode and the driving electrode at the critical equilibrium state.

The switch beam can be equivalently regarded as rigid due to minimal deformation, and its mechanical properties can be described by the linear elastic coefficient [26]. The expression for the restoring force experienced by the upper electrode can be formulated as

(3) $$F_{0}=k\left(g-g_{0}\right)$$

(4) $$k_{1\ldots 7}=\frac{EWT^{3}}{4L^{3}}$$

(5) $$\frac{1}{k}=\frac{1}{k_{1}}+\ldots +\frac{1}{k_{7}}$$

Here, k is the equivalent elasticity coefficient of the upper electrode, g represents the gap between the upper electrode and the driving electrode, and E, W, T, and L correspond to the Young’s modulus, width, thickness, and length of the cantilever, respectively. When the upper electrode is drawn to two-thirds of the distance g between the upper and lower electrodes, the electrostatic force and restoring force achieve a critical equilibrium state, from which the drive voltage formula can be derived:

(6) $$V_{\mathrm{pull}\text{-}\mathrm{down}}=V\left(\frac{2}{3}g\right)=\sqrt{\frac{8k}{27\varepsilon_{0}A}g^{3}}$$

By examining Equation (6), to design a switch with the desired low drive voltage, we crafted a folded beam with a low k value. The corresponding switch response time, considering the presence of damping, is [27]

(7) $$\;t_{s}\approx \frac{9V_{pull\text{-}down}^{2}}{4\omega_{0}QV_{s}^{2}}$$

where, $V_{s}$ is generally taken as $1.3V_{pull\text{-}down}∼1.4V_{pull\text{-}down}$, *Q* is the quality factor, and the resonant frequency is $w_{0}=\sqrt{k/m}$.

While optimizing the cantilever beam to achieve lower actuation voltages and response times, the ability of the beam to withstand stress under the optimized geometry should also be considered. Stress on the cantilever beam begins to develop as deformation progresses, and exceeding a certain threshold will lead to beam failure. Equation (8) provides the critical stress value [28].

(8) $$\sigma_{cr}=\frac{\pi^{2}{\mathrm{ET}}^{2}}{3L^{2}\left(1-v\right)}$$

where v is the material’s Poisson’s ratio and $\sigma_{cr}$ is the critical stress value.

The self-actuating voltage can be calculated through the following steps:

(9) $$V_{\mathrm{self}}=\sqrt{\frac{2k\left(g_{0}-g\right)g_{0}^{2}}{\varepsilon_{0}A}}$$

The maximum power it can withstand can be calculated using the following equation:

(10) $$P_{pull\text{-}down}=\frac{1}{4}\frac{V_{self}^{2}}{Z_{0}}$$

where $Z_{0}$ is the characteristic impedance.

## 3. Results and Discussion

### 3.1. Low Voltage and Rapid Response

**Figure 3:**
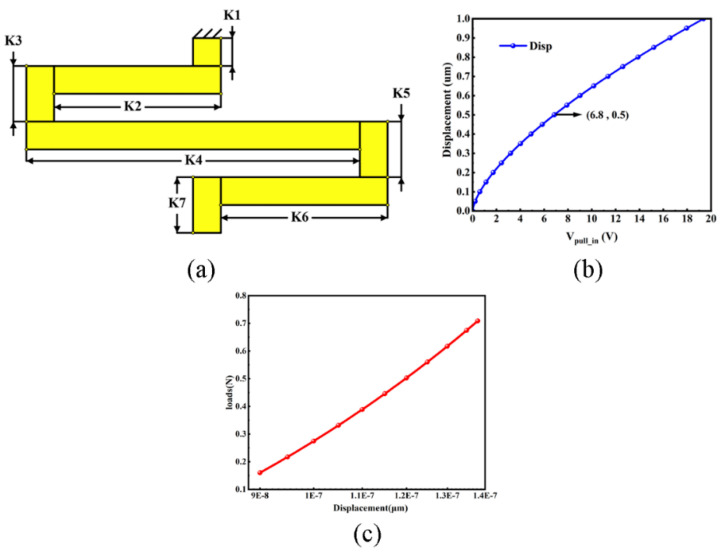
(**a**) Designed folded beam; (**b**) Pull-down voltage versus displacement relation; (**c**) Load–displacement relationship diagram.

This section details the design and optimization of the switch cantilever beam and the associated upper electrode. To reduce the driving voltage in MEMS switches, engineers often adopt one fundamental approach: diminishing the spring constant of the cantilever beam. The spring constant k for multi-cantilevers can be derived from Equation (5). It is analytically determined that the overall value of k is significantly influenced by the minimal value of $k_{1\ldots 7}$. Thus, it is imperative to minimize this value. Another point of consideration is that the pull-in voltage is directly proportional to the material’s Young’s modulus. Following a thorough analysis, gold has been chosen as the beam material due to its excellent conductivity, chemical stability, and low contact resistance for the electrical contacts, which reduces reactions with ambient air or humidity. It is worth noting that while gold offers many advantages, it also has some limitations such as creep and low softening/melting temperatures, which can affect power handling and long-term reliability. Despite these challenges, the overall benefits of using gold in our design, particularly its excellent conductivity and low contact resistance, make it a suitable choice for the beam material. The designed folded-beam structure is depicted in Figure 3a, whereas Figure 3b elaborates on the relationship between the drive voltage and displacement for the proposed RF MEMS switch. The relationship between the pull-in force and displacement can be seen in Figure 3c. The lengths of each beam segment are provided in Table 2, where each portion of the folded beam maintains a consistent width of 1 μm. According to Equations (2) and (3), the cantilever beam undergoes pull-in at a force of 1.3 μN. COMSOL simulation results indicate that the switch pulls in at a force of 1.2 μN. From Equations (2)–(7), it can be derived that the cantilever beam’s spring constant (*k*) is 0.8254, which corresponds to a driving voltage of 6.8 V and a response time of 2.28 μs. From Equations (8) and (10), the self-actuation power of the proposed switch is calculated to be approximately 1.58 W. The contact force is about a kilo μN. From these metrics, our curved folded-beam ohmic RF MEMS switch design boasts a diminished spring constant, excelling in pull-down voltage and response time characteristics.

**Figure 4:**
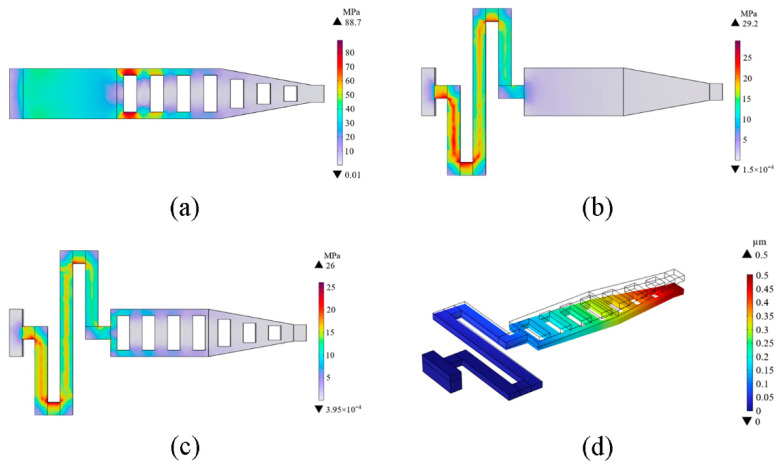
COMSOL stress simulation. (**a**) Regular perforated beam; (**b**) Improved unperforated beam; (**c**) Improved perforated beam; (**d**) COMSOL simulation in pull-down state.

A finite element simulation analysis was executed using COMSOL Multiphysics 6.0 software to evaluate the structure’s reliability further. As portrayed in Figure 4, materials with a high Poisson’s ratio and thermal expansion coefficients are optimal for control voltage minimization. Consequently, gold was chosen as the material for the upper electrode in this study. Given the short length of the designed cantilever beam, the critical stress value is relatively high, and through calculation, this value is found to be 800 MPa. The simulation results highlight that the root of the cantilever beam, where it connects to the fixed end, is the primary location for stress concentration. Due to significant bending stress in this region, the root of the cantilever beam endures the maximum bending and shear stress when a voltage is applied. The simulation images show that the stress is highest in the root area, represented in red. Similarly, the corner regions of the cantilever beam also experience stress concentration due to geometric discontinuities, exhibiting higher stress when subjected to forces. These regions are shown with high stress values, with colors approaching red in the simulation. By comparing Figure 4a with Figure 4c, it becomes evident that our proposed curved folded-beam design significantly reduces stress concentration compared to conventional straight beams. This enhances the cantilever beam’s reliability and stability [28]. Such design advantages facilitate faster activation of RF MEMS switches at reduced voltage conditions. The comparison between Figure 4b and Figure 4c underscores that the perforation method for the upper electrode not only decreases the damping coefficient and augments switching speed but also alleviates post-deposition residual stress and reduces Young’s modulus. These enhancements further amplify the switch speed and diminish the requisite driving voltage, leading to optimized performance of the RF MEMS switch. Due to these technical refinements, the beam’s peak stress has been curtailed to 26 MPa, thereby ensuring heightened reliability and stability of the switch [29]. The simulated pull-down state of the cantilever beam in COMSOL is showcased in Figure 4d, validating the switch’s performance under operational conditions, indicating its capability to pull down by 0.5 μm.

### 3.2. Key Factors Affecting RF Performance

**Figure 5:**
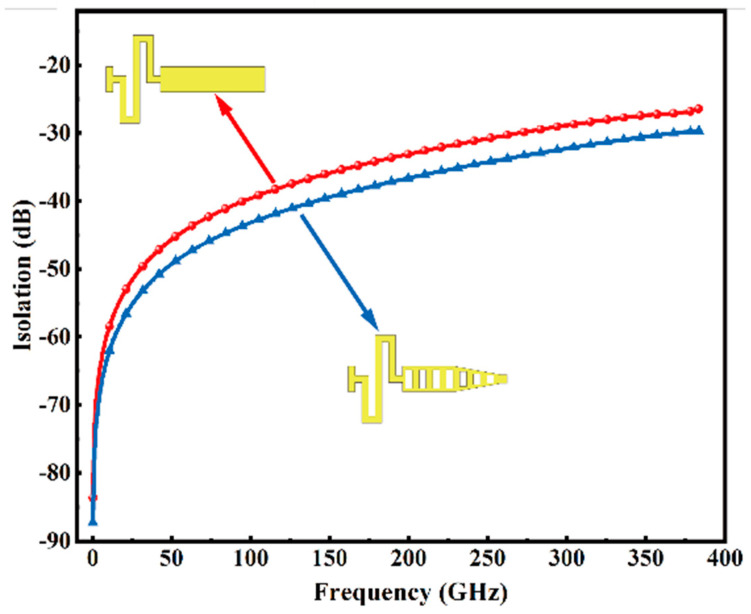
Comparison curve of the isolation of the flat plate and the electrode proposed in this research.

In RF MEMS switch applications, isolation is a paramount parameter for assessing switch performance. When the operational frequency of the switch reaches certain thresholds, isolation may decline significantly, jeopardizing adequate signal segregation. In addressing this challenge, our study introduces a novel upper electrode structure, informed by an analysis via Equation (1). The strategy predominantly entails narrowing the width of the upper electrode’s tip, consequently reducing its coupling area with the signal line, thereby augmenting the impedance during the switch’s off state. This approach enables our design to dramatically mitigate RF signal coupling from input to output, leading to superior isolation performance. Additionally, our switch design incorporates a graded structure to curtail RF performance degradation. This design aims to maintain high isolation levels while diminishing signal loss during transmission, thereby optimizing the switch’s overall RF performance. Isolation levels between the conventional straight electrode and our innovative upper electrode design are compared in Figure 5. The results highlight that our upper electrode design offers superior isolation under high-frequency conditions. By contrasting various electrode designs, this research offers potent design and theoretical foundations for optimizing isolation in high-frequency applications of RF MEMS switches.

**Figure 6:**
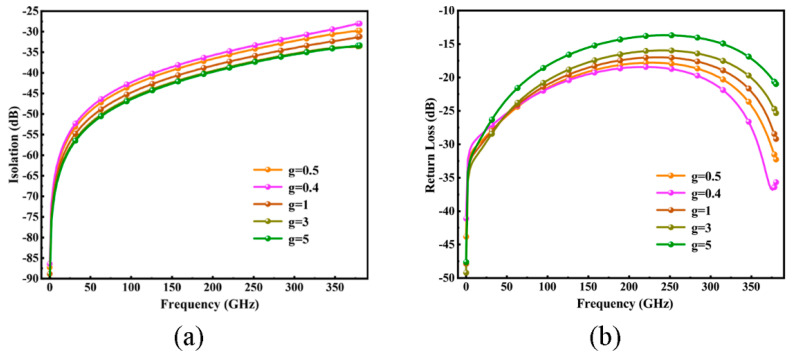
Switch (**a**) return loss and (**b**) isolation curve under different *g* values.

The air gap, denoted as g, is a critical determinant of the switch’s RF and mechanical properties. The off-state capacitance is a pivotal metric for ohmic-contact RF MEMS switches in gauging a switch’s signal isolation capability. It is discerned that g is the predominant factor prompting variance in off-state capacitance under uniform switch parameters and material conditions. The influence of air gaps on isolation and return losses is depicted in Figure 6. For our study, considering that the trade-off of enlarging the gap is the increase in pull-in voltage, we opted for a g value of 0.5 μm.

**Figure 7:**
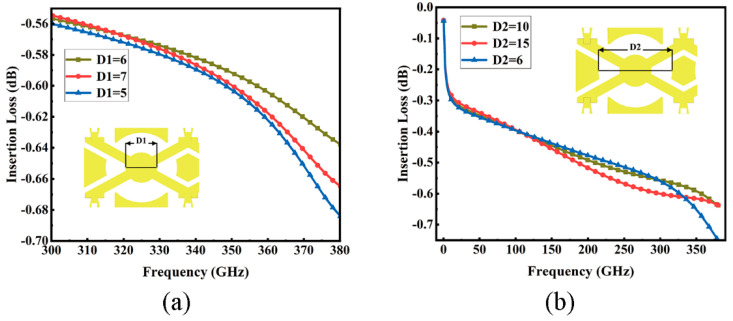
Power divider. (**a**) *D1* optimization; (**b**) *D2* optimization.

Power dividers are crucial in multi-throw switches, where an adept design can maximize signal conservation. In a specific study, RF MEMS switches based on symmetrical circular topology, notably SP7T and SP11T, have been proposed. These switches, with compact dimensions of merely 0.61 mm × 0.61 mm, are significantly smaller than conventional CMOS Si-on-insulator switches. Another study presented a radiating MEMS power divider/combiner design that enables equidistant current distribution across multiple switches, offering the merits of bandwidth, low loss, and compactness [30]. Lastly, research has showcased a generic methodology to assess multi-port centric feed circular microstrip disk power divider/combiner circuits, grounded in planar circuit techniques, deploying two-dimensional Green’s functions of circular sections [31]. In our work, we adopt a circular topology for the power divider to diminish insertion loss and condense switch size, followed by size optimization. The insertion loss curve of Figure 7 establishes the final dimensions: *D1* at 6 μm and *D2* at 10 μm. Such optimization further trims insertion loss while preserving switch compactness, enhancing the performance and efficiency of multi-throw switches.

### 3.3. Current Density Distribution and S-Parameters

**Figure 8:**
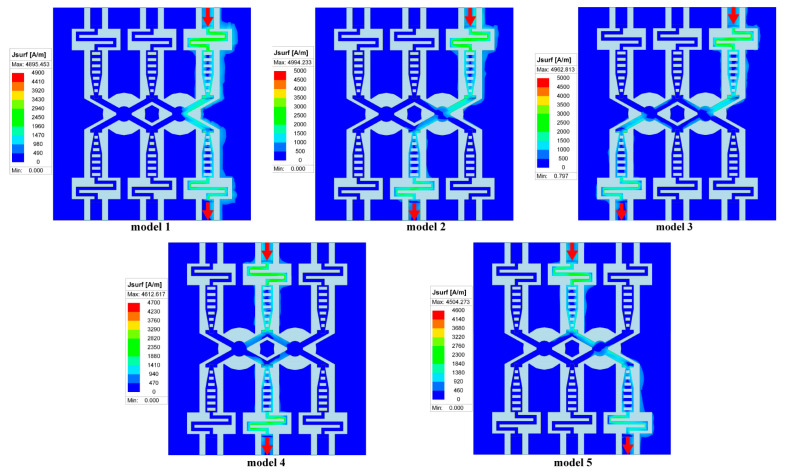
Representative current density patterns of the switch in various operational modes.

Finally, to visually demonstrate the performance of our optimized switch, we present both the current density plot and the S-parameter graph. The current density distribution of the switch under its five typical operating states is illustrated in Figure 8. Distinctly, the red arrows unambiguously represent the direction of signal transmission; for instance, Model 1 signifies the signal transition from the input at port 1 to the output at port 4, with other models following a similar pattern. It should be underscored that, given the multitude of the switch’s operating modes, symmetrical models are omitted for clarity. In the off state, there is a notable decline in current density at the disconnection region, reaching an almost negligible value. This minimal current characteristic endows the switch with superior isolation capabilities, ensuring the realization of high isolation levels. Conversely, the current density manifests a consistent distribution in the on state, facilitating a seamless current flow throughout the switch’s entire structure. This not only ensures minimal insertion loss but also maintains signal integrity. Furthermore, this consistent current distribution substantially reduces the risk of hotspot formation and localized overheating, enhancing the switch’s durability and operational stability.

**Figure 9:**
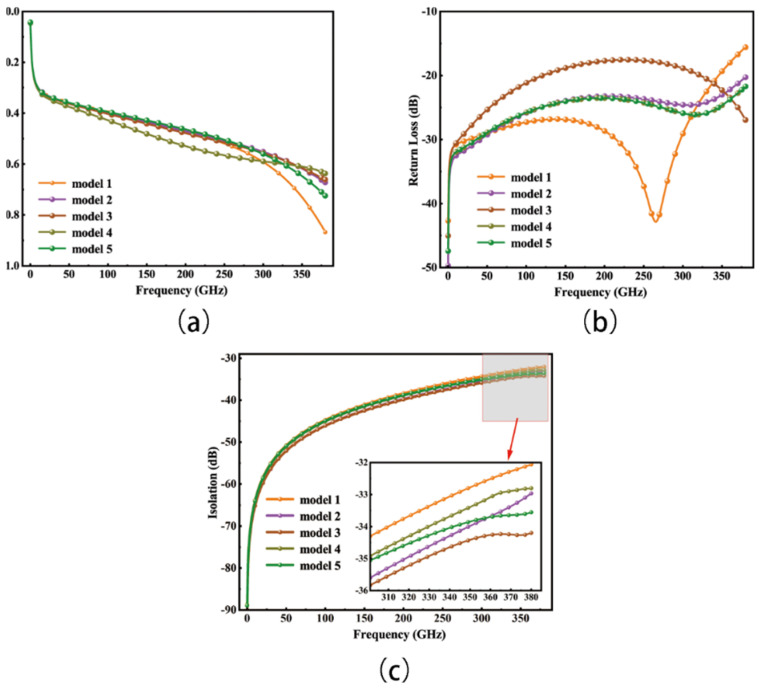
Switch S-parameters. (**a**) Insertion loss; (**b**) Isolation (average isolation across ports); (**c**) Return loss.

The Scattering parameters, commonly known as S-parameters, serve as essential tools in describing and assessing the electrical behavior of the switch in terms of its reflection and transmission properties. The S-parameter characteristics of the switch are presented in Figure 9, with Figure 9a depicting an insertion loss below 0.66 dB in the on state, Figure 9b showing that, for model 1, the return loss is less than −15 dB across the entire working frequency range, reaching close to −43 dB around 265 GHz, while the other modes have a return loss of less than −17.5 dB across this range, and Figure 9c showcasing an isolation of more than 32 dB in the off state. These results underscore the switch’s prowess in ensuring reduced signal attenuation, effective reflection control, and robust isolation in high-frequency and multifrequency scenarios. These performance metrics convey the ability of the signal to traverse the switch with minimal losses, ensuring negligible interference between ports. Such an attribute substantially reduces the potential for signal crosstalk and interference, enhancing overall signal transmission quality.

**Figure 10:**
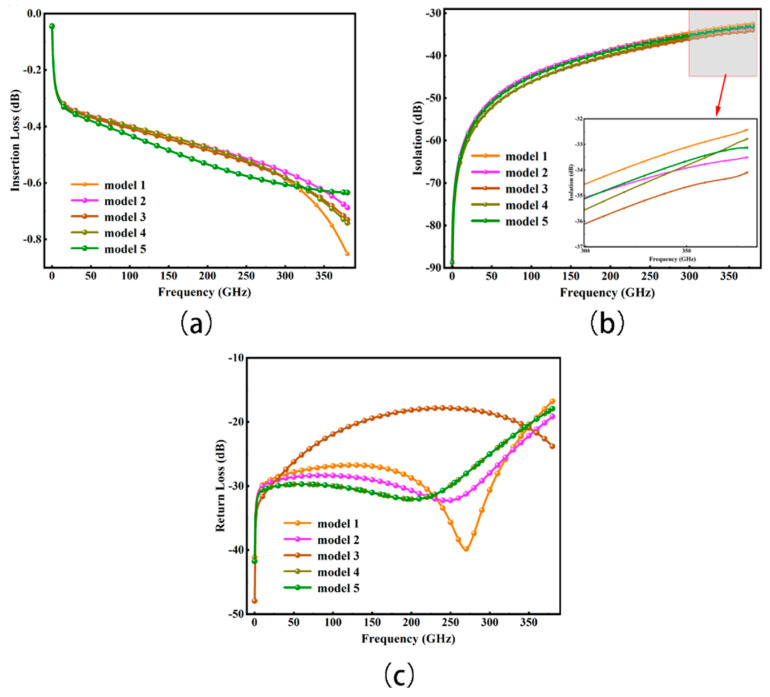
Switch S-parameters with packaging. (**a**) Insertion loss; (**b**) Isolation (average isolation across ports); (**c**) Return loss.

The suggested packaging employs BCB glue and high-resistance silicon. Our simulation results indicate that the performance does not significantly fluctuate after packaging, as shown in Figure 10.

Compared with prior research, Table 3 denotes that our proposed switch manifests significant advantages in multiple facets. Specifically, our switch offers a broader bandwidth, lower driving voltage requirements, faster response times, and superior signal isolation, rendering it a more competitive solution.

## 4. Conclusions

This research presents a 3P3T RF MEMS switch operating across frequencies from DC to 380 GHz. ANSYS HFSS 2023 R2 simulations validate its exceptional RF performance. Achieving insertion losses below 0.66 dB across such a wide frequency band and up to high frequencies is particularly commendable. Moreover, maintaining low insertion loss while elevating isolation to over 32 dB demonstrates the advanced level of our switch. The switch’s six-port configuration enhances signal modulation capabilities. COMSOL simulation indicates an optimized maximum stress of 26 MPa for the switch, achieved through the innovative power-up stage and folded-beam design. This has led to a reduced actuation voltage of 6.8 V and a quick response time of 2.28 μs, surpassing most RF MEMS switches. In our study, we also provide S-parameter plots and current density plots under typical operating conditions, further substantiating the switch’s robust RF performance. Given its balanced efficiency and reliability, this switch shows potential for driving advancements in 6G communications, phased-array antennas, and satellite communications.

## Figures and Tables

**Table 1:** Dimensions of the designed switch.

Parameters	Values (µm)
CPW Size	2/3.75/2
CPW Thickness	2
Top Electrode Thickness	0.5
Top Electrode Length	15.5
Top Electrode Width	3.75
Gap	0.5
Overall Size	63 × 68

**Table 2:** Dimensions of different parts of the proposed folded beam.

*K*	*K*1	*K*2	*K*3	*K*4	*K*5	*K*6	*K*7
*L* (μm)	1	6	3	12	3	6	2

**Table 3:** Comparison of performance parameters of the proposed switch with previous works.

Ref.	Frequency (GHz)	Isolation	Insertion Loss	Return Loss	Drive Voltage	Switching Time
[32]	220–280	>17 dB	<4.2 dB	>11 dB	-	-
[21]	140–480	>12 dB	<3 dB	>10 dB	55 V	-
[22]	DC-480	>13 dB	<1.37 dB	-	-	500 ps
[24]	DC-150	>20 dB	<0.18 dB	>23 dB	20 V	3.2 μs
[33]	100–200	>19 dB	<0.8 dB	>20 dB	23.17 V	-
This Work	DC-380	>32 dB	<0.66 dB	>15 dB	6.8 V	2.28 μs

## Data Availability

The data that support the findings of this study are available from the corresponding author upon reasonable request.
